# Sleep characteristics of Iranian people and their effects on daytime functioning: a population-based study

**DOI:** 10.1038/s41598-022-07686-3

**Published:** 2022-03-10

**Authors:** Samaneh Akbarpour, Khosro Sadeghniiat-Haghighi, Alireza Delavari, Shahnam Arshi, Mohammad Alirezaei, Faezeh Aghajani, Amin Nakhostin-Ansari, Zahra Banafsheh Alemohammad, Ania Rahimi-Golkhandan, Arezu Najafi

**Affiliations:** 1 Sleep Breathing Disorders Research Center, Tehran University of Medical Sciences, Tehran, Iran; 2 Occupational Sleep Research Center, Baharloo Hospital, Tehran University of Medical Sciences, P. O. BOX: 133 99 73 111, Tehran, Iran; 3 Digestive Disease Research Institute (DDRI), Shariati Hospital, Tehran University of Medical Sciences (TUMS), Tehran, Iran; 4 Infectious Diseases and Tropical Medicine Research Center, Shahid Beheshti University of Medical Sciences, Tehran, Iran; 5 Social Determinants of Health Research Center, Shahid Beheshti University of Medical Sciences, Tehran, Iran; 6 School of Medicine, Tehran University of Medical Sciences, Tehran, Iran; 7 Research Development Center, Arash Women’s Hospital, Tehran University of Medical Sciences, Tehran, Iran; 8 Sports Medicine Research Center, Neuroscience Institute, Tehran University of Medical Sciences, Tehran, Iran

**Keywords:** Health care, Medical research

## Abstract

Sleep characteristics vary between populations. Detrimental sleep habits have cognitive consequences leading to daytime functioning debilitation. Until now no study has been done to investigate sleep characteristics in Iran thoroughly. In this study, we aimed to evaluate Iranians’ sleep characteristics and their association with daytime functioning. We conducted a population-based study from January 2017 to May 2019 on people more than 18 years old who lived in 11 urban destricts and 3 rural areas of Tehran, Iran. We randomly selected the participants using a multistage random stratified clustered sampling method. We obtained the participants’ demographic and anthropometric characteristics and details of bedtime, sleep duration, sleep onset latency, wake-up time and sleep impact on daytime functioning. Logistic regression model was used to assess the relationship between sleep characteristics) and daytime functioning. In total, 1830 people with a mean age of 40.83 years participated in the study. The gender distribution of the participants was even, and 70.98% of them were married. After adjusting for age and sex, the following three factors had a significant impact on daytime functioning: bedtime, sleep onset latency, and sleep duration. (OR = 1.12, *P* < 0.038, OR = 1.01, *P* < 0.011, and OR = 0.99, *P* = 0.01, respectively). We also found that longer sleep onset latency (*P* = 0.004) and shorter sleep durations (*P* = 0.029) significantly interfere with daytime functioning. Iranians’ sleep characteristics, especially their sleep duration and sleep onset latency, are associated with their daytime function. Interventions on people’s sleep hygiene are warranted to promote healthier sleep behaviors among Iranians, considering the high impact of current sleep characteristics on their daily lives.

## Introduction

The National Sleep Foundation recommends between 7 and 9 h of sleep during the day for adults^1^. However, despite regulatory processes, several medical, neurological, and psychological conditions may lead to sleep deprivation^2^, defined as decreased performance due to inadequate sleep^3^. Also, multiple factors, including age and gender, may affect sleep characteristics^4^, and different populations may have different sleep features^5^.

The prevalence of sleep disorders has increased in recent years^6^, and about 9 to 15% of adults suffering from chronic sleep disturbances report daytime consequences of their poor quality sleep^7^. In a large–scale study in the United States (US), 34.8% of adults reported short sleep duration (< 7 h). However, the prevalence of short sleep duration varied considerably across different states, as in some states, the prevalence of short sleep duration was about 54%^8^. Other demographic characteristics are also associated with sleep deprivation; for example, sleep deprivation is more prevalent in some occupations, such as healthcare workers^9^. Also, sleep characteristics vary in different populations, and findings in one country may not be applicable to other populations^5^.

Sleep deprivation may cause fatigue, daytime sleepiness, and memory impairment. Anxiety, depression, and cardiovascular diseases are among disorders associated with sleep deprivation^2^. Studies have shown that insomnia increases the risk of car accidents by 2.5 to 4.5 times^10^. Also, an international study revealed that about 7% of people with insomnia had at least one episode of a car accident in the past year prior to the study. Furthermore, the car accidents were associated with several sleep problems, including difficulty initiating sleep, early morning awakening, non-restorative sleep, and poor sleep quality^11^. Sleep deprivation may lead to decreased productivity, and people with less sleep duration lose more productivity time^12,13^. Sleep characteristics other than sleep duration, such as rise time and bedtime, may also contribute to people’s daily functioning. For instance, irregular bedtime may lead to decreased daytime functioning^14^.

Altogether, sleep characteristics differ between populations, and sleep problems have several negative cognitive, psychological, and functional impacts. Several studies investigated the sleep characteristics of the Iranian population. However, most of them focused on specific groups such as students, drivers, and people with health conditions^15–18^, and there is limited evidence on the sleep characteristics of the general Iranian population. Also, no study has evaluated the association between sleep characteristics and daytime functioning in Iran. To consider this matter, in this large-scaled study, we evaluated the sleep characteristics of Iranian people and evaluated its association with daytime functioning.

## Materials and methods

### Study design

This population-based study was conducted from January 2017 to May 2019 in Tehran, the capital of Iran. The study was approved and funded by the Tehran University of Medical Sciences’ Ethics Committee (IR.TUMS.VCR.REC.1397.236). We confirm that all methods were performed in accordance with the relevant guidelines and regulations.

To describe the Iranian population’s sleep characteristics, we conducted a population-based study on 2000 participants from the center of Tehran province, Tehran, chosen by the multistage random stratified clustered sampling method (1830 participants were included in the analysis, 91% Response rate). Tehran is Iran’s capital city with about 12 million inhabitants and is divided into 22 districts administratively. The cluster sampling method was used to select a representative sample of Tehranian adults aged > 18 years old.

We randomly selected 11 districts in urban areas and 3 rural areas of Tehran. The clusters, each consisting of 10 males and ten females, were taken from each district proportional to Tehran’s total population, and in total, we had about 100 clusters. After that, we randomly selected a healthcare center in each of the 11 urban districts and three rural areas. We randomly selected a household affiliated with the selected healthcare center for each cluster. We had two trained interviewers who went for the interviews in coordination with health care center staff. They went to the address of the selected household and gave explanations about the study goals and objectives. If they agreed to participate in the study, one male and one female who met the eligibility criteria, being Iranian and 18 years or older, were randomly selected from that household and were interviewed. After that, they went to the household on the right side of the selected household and repeated the same procedure. Interviewers continued sampling until they recruited 20 people for each cluster using the explained procedure.

One hundred seventy participants who had severe physical or psychological disabilities or who were taking sleep medications were not entered in the final analysis.

### Measurements

Individuals after completing inform consent forms were interviewed by trained interviewers as main staffs in the study. Each interviewer staff passed a training workshop regarding the study aim, protocol and communications in households in each region for data collection.

The staff filled a questionnaire by interviewing each participant face-to-face. The questionnaire contained demographic (age, gender, marital status, and educational status) and anthropometric data (weight, height, and body mass index^19^, measured by interviewers using Matheo Digital Scale manufactured by Germany and NOVA NTT 3016 m manufactured by Iran), and questions regarding sleep characteristics (Bedtime, sleep onset latency, usual wake-up time, sleep duration on weekdays, sleep duration in weekends)^20^ and sleep satisfaction (problems with waking up too early, satisfaction with current sleep pattern, and interference of sleep problems with daily functioning) in the last month^21^. We also asked them whether they think their sleep problems affect their daytime functioning or not.

We used Pittsburgh Sleep Quality Index (PSQI) to evaluate the participants’ sleep characteristics. PSQI has 24 questions, 19 questions are self-rating questions, and a roommate or a bed partner scores the remaining five^20^. PSQI has been translated to Persian and proved to be a reliable, valid, sensitive, and specific tool evaluating sleep quality in Iranian People^22^. We included the first four questions of PSQI in our study evaluating bedtime, sleep onset latency, wake-up time, and sleep duration on weekdays and weekends. We used the crude numbers reported by the patients in our data analysis.

Insomnia Severity Index (ISI) is another questionnaire used for the evaluation of insomnia. This questionnaire consists of seven items, and each can be answered from 0 to 4 on a Likert scale where higher scores were indicative of more severe sleep problem^21^. ISI has been translated to Persian and was shown to be a reliable and valid tool evaluating insomnia severity in Iranian people^23^. In our study, we used three ISI questions evaluating problems waking up early, satisfaction with current sleep patterns, and interference of sleep problems with daytime functioning. We categorized the subjects based on the reported severity of the sleep problems and if they had daytime dysfunction due to sleep problem.

### Statistical analysis

Approximately, 4.17% of covariate data cells were missed and we used a single imputation method based on regression model for estimation of missing information by using mice package in R software. We used complex survey methods to enter cluster and sampling weight. All data analyses were weighted based on the 2011 Iran population census in Tehran based on gender and age categories in survey analysis.

To describe continuous and categorical variables weighted means and Standard Error (SE) and the weighted percentage were reported, respectively based on survey analysis. To compare means between two or more independent groups student t-test and one-way ANOVA were used, respectively and to qualitative variables between two or more groups x-square test was used.

In order to assess the relationship between sleep characteristics (as main exposures) and daytime functioning (as interesting outcome) logistic regression model was used and crude and age and sex-adjusted odds ratios were reported. To assess the trends of prevalence of interfere with daytime functioning, we generated quartile boundaries for the distribution of sleep characteristics and determined the prevalence of them in each quartile.

All data analyses were done by STATA software (R for imputation), and all p-values were 2-tailed and p-value ≤ 0.05 was considered significant.

## Results

In total, 1830 people (907 men and 923 women) with a mean age of 40.83 ± 0.08 years participated in the study. Response rate was 91%. Most of the sample population lived in the urban areas (94.37%) and were married (70.89%). Table 1 demonstrates the demographic and anthropometric characteristics of the studied population.

**Table 1:** Demographic and anthropometric characteristics of study participants (N = 1830).

Variable		Mean ± SD/ Percentage
Age		40.83 ± 0.08
BMI		26.94 ± 0.28
Neck Circumference		37.88 ± 0.33
Gender	Male	50.01
	Female	49.98
Educational status	Illiterate	26.15
	Diploma or lower	38.26
	Higher than diploma	35.57
Marital status	Single	23.40
	Married	70.89
	Divorced	5.70
Living area	Urban	94.36
	Rural	5.63

*BMI* Body Mass Index.

**Figure 1:**
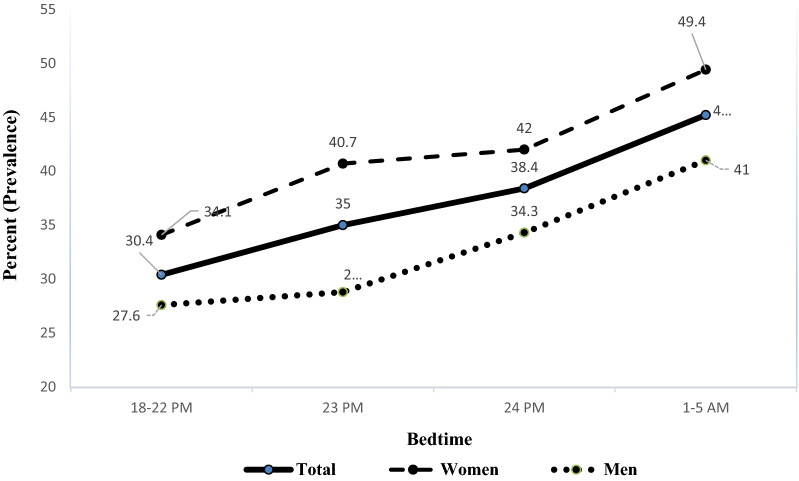
Bedtime trend in based on gender among study population.

**Figure 2:**
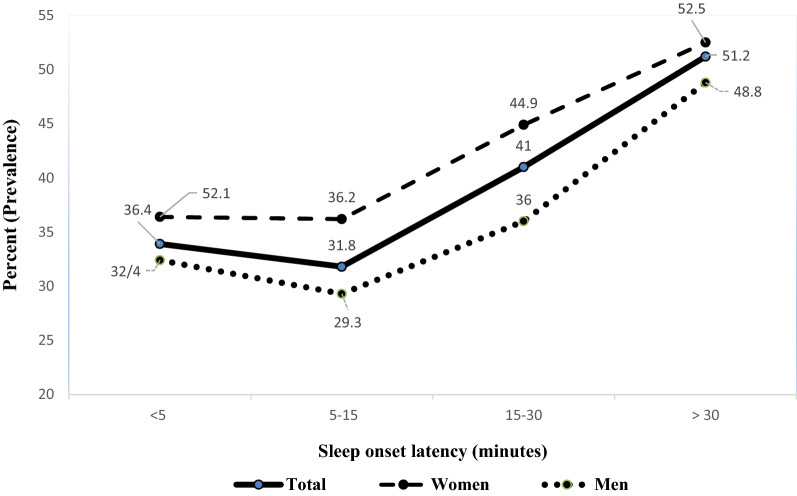
Sleep onset latency trend based on gender among study population.

**Figure 3:**
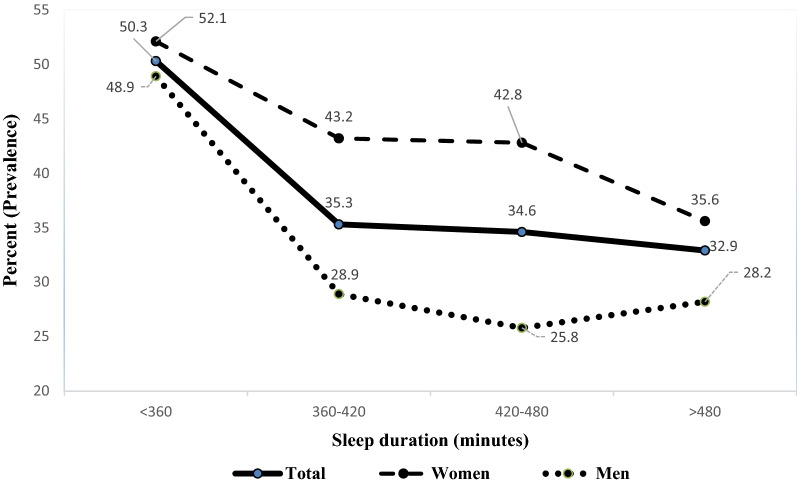
Sleep duration trend based on gender among study population.

**Figure 4:**
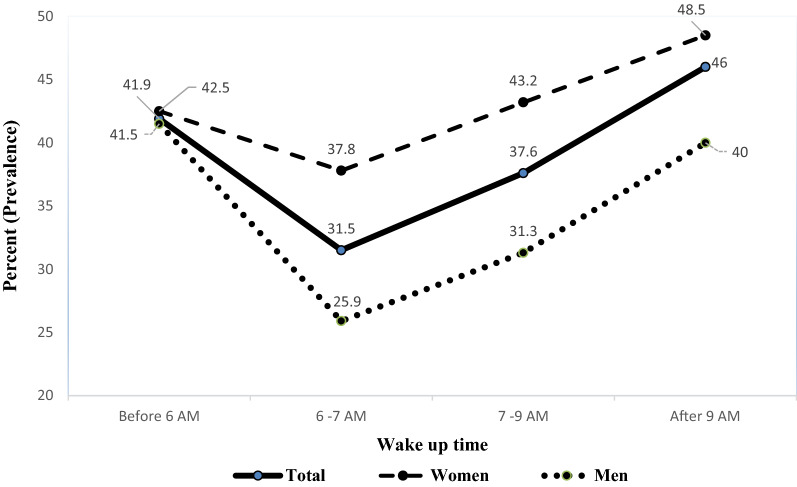
Wake up time trend based on gender among study population.

Disturbution of bedtime, sleep onset latency, sleep duration, and wake-up time are depited in Figs. 1–4 among men, wemon, and whole population, respectively. We found that older and married participants with higher BMI, illiterates, and those who lived in urban areas had more problems waking up too early (*P* < 0.05). Also, participants with higher BMI and females we more satisfied with their current sleep pattern (*P* < 0.05). We found that sleep problems significantly interfered with the daytime functioning of people with higher BMI and people living in urban areas (*P* < 0.05). Sleep problems negatively impacted the daytime functioning of the urban population and divorced participants (*P* < 0.05). Table 2 shows the association between demographic and anthropometric characteristics and sleep problems.

**Table 2:** Demographic and anthropometric characteristics of participants with different sleep problems.

		Problems waking up too early				Satisfaction from current sleep pattern				Interfere with daytime functioning				The negative impact of sleep problems on daytime functioning*			
		None	Mild& moderate	Severe & very severe	*P*-value	Very dissatisfied & dissatisfied	Moderately Satisfied	Satisfied & very satisfied	*P*-value	Not at all or a little	Somewhat	Much and very much	*P*-value	Yes		No	*P*-value
		**N = 1139**	**N = 566**	**N = 125**		**N = 1191**	**N = 290**	**N = 349**		**N = 1106**	**N = 307**	**N = 417**		**N = 1123**		**N = 707**	
Age		38.61 ± 0.38	42.84 ± 0.67	48.26 ± 0.92	** < 0.0001**	40.40 ± 0.34	42.06 ± 1.09	41.23 ± 0.84	0.330	40.79 ± 0.41	40.10 ± 1.12	41.45 ± 0.94	0.671	41.58 ± 0.60		40.23 ± 0.48	0.219
BMI		26.63 ± 0.32	27.18 ± 0.27	28.21 ± 0.72	**0.001**	26.66 ± 0.28	27.26 ± 0.35	27.55 ± 0.40	**0.011**	26.59 ± 0.31	27.27 ± 0.37	27.47 ± 0.27	**0.009**	27.21 ± 0.38		26.72 ± 0.28	0.188
Neck Circumference		37.79 ± 0.47	37.90 ± 0.26	38.43 ± 0.46	0.630	37.66 ± 0.27	37.41 ± 0.39	38.85 ± 1.11	0.325	37.56 ± 0.28	38.83 ± 1.33	37.87 ± 0.44	0.270	37.85 ± 0.31		37.85 ± 0.31	0.921
Gender	Male	48.97	52.19	48.13	0.445	53.59	44.45	43.35	**0.002**	52.45	45.92	47.65	0.274	44.74		54.25	0.278
	Female	51.02	47.80	51.86		46.40	55.54	56.64		47.54	54.07	52.34		55.25		45.74	
Education status	Illiterate	23.15	28.65	37.13	**0.034**	25.67	27.05	26.94	0.762	27.96	22.53	24.82	0.142	22.39		29.18	0.141
	Diploma or lower	41.01	34.76	33.45		38.44	35.27	39.90		36.55	36.73	43.06		38.82		37.81	
	Higher than diploma	35.83	36.57	29.40		35.87	37.66	33.15		35.47	40.73	32.10		38.78		33.00	
Marital status	Single	27.56	19.70	9.75	** < 0.0001**	24.31	20.37	22.87	0.713	24.87	22.85	20.79	0.659	36.68		63.31	** < 0.0001**
	Married	67.09	75.17	79.92		70.05	74.51	70.76		69.39	70.89	74.15		45.57		54.42	
	Divorced	5.33	5.12	10.90		5.63	5.10	6.36		5.73	6.52	5.05		51.82		48.17	
Place of living	Urban	91.81	97.60	98.81	**0.008**	93.35	94.89	96.99	0.075	92.14	96.97	97.31	**0.0459**	45.58		54.41	** < 0.0001**
	Rural	8.18	2.39	1.18		6.64	5.10	3.00		7.85	3.02	2.68		15.25		84.74	

Data are reported weighted percent for all variables except age, BMI and neck are reported as weighted mean and standard error.

Mean bedtime and wake-up time were 24.06 ± 1.45 and 7.53 ± 0.07, respectively, with a mean sleep duration of 431.31 ± 5.59 min. We found that older and illiterate adults and divorced people go to bed sooner (*P* < 0.05). Younger, thinner males, higher educated, and single adults, and those with shorter neck circumferences woke up later (*P* < 0.05). We found that males have a shorter sleep onset latency than females (22.6 vs. 35.54, *P* < 0.0001), but there was no significant association between other demographic and anthropometric characteristics and sleep onset latency (*P* > 0.05). Younger adults, females, and urban residents had longer sleep duration on workdays (*P* < 0.05). On the weekends, younger adults, those with lower BMI and shorter neck circumferences, higher educated, and single adults had a longer sleep duration (*P* < 0.05). Table 3 shows the association between sleep and demographic and anthropometric characteristics.

**Table 3:** Demographic and anthropometric characteristics of participants with different sleep characteristics.

		Bedtime		Waking-up time		Sleep onset latency (min)		Sleep duration (min)		Weekend sleep duration (min)	
		Mean (SD)/ r	*P*-value	Mean (SD)/r	*P*-value	Mean (SD)/r	*P*-value	Mean (SD)/r	*P*-value	Mean (SD)/r	*P*-value
Age (year)	> 50	23.68 ± 0.06	** < 0.0001**	6.98 ± 0.12	** < 0.0001**	29.58 ± 2.92	0.746	413.99 ± 6.16	** < 0.0001**	453.76 ± 7.39	** < 0.0001**
	< 50	24.01 ± 0.08		7.75 ± 0.08		28.86 ± 1.37		437.98 ± 6.16		519.88 ± 6.25	
BMI (kg/m^2^)	> = 35	23.93 ± 0.11	0.555	7.32 ± 0.06	**0.003**	31.48 ± 4.25	0.532	431.97 ± 12.49	0.958	471.02 ± 12.00	**0.003**
	< 35	23.99 ± 0.07		7.61 ± 0.08		28.90 ± 1.65		431.35 ± 5.58		503.70 ± 6.06	
Neck Circumference (cm)	≥ 40	24.00 ± 0.09	0.674	7.16 ± 0.08	**0.001**	24.78 ± 2.38	0.045	415.88 ± 7.31	0.003	489.72 ± 7.07	**0.010**
	< 40	23.96 ± 0.07		7.66 ± 0.09		30.22 ± 1.80		436.01 ± 6.02		504.95 ± 6.63	
Gender	Male	23.95 ± 0.09	0.429	7.14 ± 0.10	** < 0.0001**	22.60 ± 1.53	** < 0.0001**	416.18 ± 8.32	** < 0.0001**	498.16 ± 7.96	0.361
	Female	24.00 ± 0.06		7.94 ± 0.10		35.53 ± 2.37		446.61 ± 3.91		505.30 ± 6.30	
Educational status	Illiterate	23.73 ± 0.09	**0.005**	7.23 ± 0.10	**0.003**	29.64 ± 3.42	0.478	427.04 ± 7.07	0.786	478.20 ± 8.11	**0.002**
	Diploma or lower	24.04 ± 0.08		7.84 ± 0.08		31.78 ± 2.82		440.09 ± 6.12		515.90 ± 9.71	
	Higher than diploma	24.08 ± 0.09		7.45 ± 0.10		25.71 ± 1.61		425.23 ± 6.73		503.79 ± 6.09	
Marital status	Single	24.35 ± 0.14	** < 0.0001**	8.10 ± 0.16	** < 0.0001**	32.03 ± 3.79	0.882	438.37 ± 9.88	0.134	518.30 ± 11.68	**0.002**
	Married	23.89 ± 0.06		7.40 ± 0.05		27.25 ± 1.55		430.42 ± 4.53		499.92 ± 5.22	
	Divorced	23.50 ± 0.11		6.93 ± 0.21		39.40 ± 6.31		414.88 ± 14.75		456.23 ± 11.49	
Living area	Urban	23.99 ± 0.07	0.103	7.53 ± 0.07	0.641	29.29 ± 1.75	0.195	453.86 ± 3.83	**0.003**	501.31 ± 6.44	0463
	Rural	23.75 ± 0.11		7.64 ± 0.19		25.16 ± 2.52		430.05 ± 5.93		508.79 ± 7.62	

*BMI* Body Mass Index.

After adjusting for age and sex, late bedtime (OR = 1.12, 95% CI = 1.01–1.21) and longer sleep onset latency (OR = 1.01, 95% CI = 1.02) had significant negative impacts on daytime functioning. In contrast, longer sleep duration had a reverse impact (OR = 0.99, 95% CI = 0.98–0.99). Sleep problems significantly interfered with the daytime functioning of those with longer sleep onset latency (*P* = 0.004) and shorter sleep durations (*P* = 0.029). Association between sleep characteristics and negative impacts of sleep problems on daytime functioning and interference of sleep problems with daytime functioning are shown in Tables 4 and 5, respectively.

**Table 4:** Sleep characteristics and their impact on daily functioning.

	Impact of sleep problems on daytime functioning		*P*-value	Crude odds ratio (95% CI)	*P*-value	Age and sex odds ratio (95% CI)	p-value
	Yes (n = 1123)	No (n = 707)					
Bedtime	24.10 (0.10)	23.88(0.07)	**0.048**	1.10 (0.99–1.22)	0.058	1.12 (1.01–1.23)	**0.038**
Sleep onset latency	34.35(2.07)	24.84 (1.75)	** < 0.0001**	1.01 (1.0–1.02)	**0.007**	1.01 (1.0–1.02)	**0.011**
Waking up time	7.59(0.10)	7.47(0.08)	0.375	1.03 (0.95–1.12)	0.352	1.03 (0.95–1.12)	0.384
Sleep duration	419.63 (7.62)	440.70 (4.84)	**0.009**	0.99 (0.98–0.99)	**0.023**	0.99 (0.98–0.99)	**0.010**
Weekend sleep duration	498.43(8.30)	501.87(8.41)	0.615	1.00 (0.98–1.00)	0.603	1.00 (0.99–1.00)	0.421

**Table 5:** Sleep characteristics and their interference with daytime functioning.

	Interfere with daytime functioning					*P*-value
	Not at all (n = 746)	A little (n = 360)	Somewhat (n = 307)	Much (n = 233)	Very much (n = 184)	
Bedtime	23.93 ± 0.06	23.81 ± 0.12	24.04 ± 0.07	24.15 ± 0.19	24.09 ± 0.14	0.112
Sleep onset latency (min.)	23.14 ± 2.13	29.08 ± 3.09	28.75 ± 2.46	34.56 ± 2.50	37.99 ± 3.23	**0.004**
Waking up time	7.55 ± 0.09	7.54 ± 0.12	7.57 ± 0.12	7.46 ± 0.13	7.55 ± 0.20	0.852
Sleep duration (min)	441.53 ± 5.11	436.10 ± 5.35	426.78 ± 8.76	416.04 ± 14.01	418.61 ± 14.01	**0.029**
Weekend sleep duration (min)	501.13 ± 6.05	498.37 ± 6.98	504.77 ± 10.33	501.10 ± 15.01	505.53 ± 17.12	0.767

## Discussion

We conducted this study to evaluate the sleep characteristics of Iranians and their impact on daily functioning. To the best of the authors’ knowledge, this is the first large-scale study of this matter in Iran. The mean sleep duration was 431.31 ± 5.59 min in our study, which is in the range of the recommended 7 to 9 h sleep duration by the National Sleep Foundation^1^. There is a considerable variation between countries regarding the reported sleep duration. Several studies have been conducted in Asian countries evaluating sleep problems using PSQI. In a study on Chinese people, the mean sleep duration was 454.75 ± 77.3 minutes^24^, which is higher than the sleep duration in our study. However, in similar studies in South Korea and Hong Kong, the mean sleep duration was lower than our study, with only 16.8% of Korean people sleeping more than the recommended seven hours^25,26^. There were also discrepancies in the roles of demographic characteristics such as gender in sleep duration. In Iran and China, sleep duration was higher in females; however, males slept for longer hours in Hong Kong and South Korea^24–26^. Considering the constant role of hormonal and physiological changes in females, which can affect sleep duration and sleep quality^27^, different employment statuses of females and different social contexts in communities^28^ may be the factors leadning to shorter or longer sleep durations in females compared to males in different countries. These disparities indicate different patterns in the distribution of factors affecting insomnia in populations and the need for studies to determine the at-risk people and contribution factors across communities.

We found that late bedtime impacts daytime functioning negatively (OR = 1.12, *P* = 0.038). In a study on adolescents, Wolfson et al. found that students with late bedtime habits had a bad academic performance^29^. Fernandez-Mendoza et al. evaluated the impact of chronotype on sleep behaviors and its association with daytime functioning, cognition, and mood. E chronotype people (evening type) tend to have late bedtime and impaired daytime functioning due to fatigue^30^. Our findings regarding the worse daily functioning in people who have later bedtimes may be due to several reasons; first, E-types have more irregular sleep patterns than M-types (morning type), which lead to sleepiness during the day^30,31^. Sleepiness during the day may by itself have negative impacts on daytime functioning. We also found that higher educated and younger Iranians have late bedtimes. Such sleep patterns may be troublesome as younger adults are more functional than older adults, and people with higher educational levels have more critical roles in the community. Late bedtime in these groups, as the functional part of the community, and the negative impacts of such sleep habits need to be addressed in future health policies to enhance Iranian people’s work efficacy.

We found that longer sleep onset latency is significantly associated with daytime functioning impairment (OR = 1.01, *P* = 0.011). Ohayon et al. also found that longer sleep latency is associated with sleep dissatisfaction^32^. People’s dissatisfaction and perception of their sleep may be the reason for attributing their functional impairment to sleep^33^. On the other hand, people with higher sleep onset latency are more affected by adverse stimuli, further affecting their daytime function perception^34^. Prolonged sleep onset latency happens in people with mood disturbances^35^, thus impairing their daytime functioning.

Sleep duration also affects daytime functioning. Males, older adults, and rural region residents had shorter sleep duration (*P* < 0.05). Early morning awakening was more common among older adults, reflecting in their wake-up time, as those older adults went to bed earlier and woke up in earlier hours. Our findings are in line with previous studies regarding a higher prevalence of early morning awakening in older adults^36^. In a similar study, Ferrie et al. found that people with higher job positions sleep more^37^. The association between sleep duration and daytime sleepiness is well studied in previous works, which, in turn, may have consequences such as the increased risk of road accidents^38,39^. Sleeping for less than six hours a day is associated with poor cognitive function^37^. Sleepiness and poor cognitive function may be reasons for impaired daytime functioning. At-risk groups such as males, older adults, and people living in rural areas may benefit from interventions to increase sleep duration. Treating the elderly for early morning awakening may be beneficial in this regard^40^.

In our study, most participants (65.08%) were dissatisfied with their current sleep patterns. In other countries, the prevalence of sleep dissatisfaction ranged between 8 and 18.5%^33^, significantly less than our findings. Several factors are independently associated with sleep satisfaction, including feeling refreshed, sleep calmness, ease of falling asleep, and awakening frequency^41^. As people’s perceptions of their sleep quality and satisfaction with their sleep are important factors regarding interpretation of sleep impacts on their daytime functioning^33^, future studies on the Iranian population are needed to determine and address the factors contributing to sleep dissatisfaction. In this study, higher educated people were not satisfied with their sleep. As they have more critical roles in society, their daytime functioning impairment may be more prominent.

### Limitations

We found an association between sleep characteristics and daytime functioning in the Iranian population, but due to the study’s cross-sectional design, a causal relationship cannot be interpreted from our findings. Future longitudinal studies are required to evaluate this relation further. People’s psychological characteristics are other factors affecting daytime functioning. Future studies evaluating more variables will help determine the factors affecting people’s daytime functioning and the factors mediating these effects.

## Conclusion

Iranians’ sleep characteristics, especially their sleep duration and sleep onset latency, are associated with daytime functioning. As we found high levels of sleep dissatisfaction among our participants, interventions to promote sleep hygiene are needed.
